# Uncertainty-aware federated temporal learning with explainable LLM-based coaching for privacy-preserving wearable health systems

**DOI:** 10.3389/frai.2026.1809361

**Published:** 2026-08-26

**Authors:** Duane Chembakassery, Harisankar R. Nair, J. Prassanna

**Affiliations:** School of Computer Science and Engineering, Vellore Institute of Technology (VIT), Chennai, India

**Keywords:** explainable AI, federated learning, large language models, semantic imputation, temporal learning, uncertainty modeling, wearable health systems

## Abstract

**Introduction:**

The growing use of wearable sensors enables continuous health and activity monitoring; however, challenges such as noisy data, privacy concerns, and device heterogeneity limit the effectiveness of centralized learning systems. This paper presents a privacy-first, AI-enhanced lifestyle coaching framework that integrates multi-stage signal cleaning, federated learning (FL), and explainable large language models (LLMs) with structured prompting for intelligent, decentralized health guidance. Unlike prior FL-based human activity recognition (HAR) systems, this work introduces (i) a quantitatively validated multi-stage denoising pipeline, (ii) local latent-space semantic imputation under federated constraints, and (iii) an explainable LLM-based coaching layer whose reasoning is explicitly grounded in aggregated federated activity representations rather than raw sensor data.

**Methods:**

The proposed system employs a robust preprocessing pipeline comprising Hampel filtering, adaptive wavelet–Butterworth denoising, Variational Autoencoder (VAE)-based semantic imputation, and Kalman smoothing. The cleaned signals are used to train highly efficient Deep LSTM models on-device, and local updates are aggregated using a FedProx-based framework to mitigate client drift and preserve data privacy. To ensure rigorous evaluation and eliminate temporal data leakage, strict chronological data splitting is enforced alongside a 50% overlap stride constraint.

**Results:**

The multi-stage signal cleaning pipeline successfully achieved a 7–10 dB improvement in signal-to-noise ratio (SNR). Under the strict evaluation conditions, the global federated model established a highly realistic, leak-free baseline, achieving an average global accuracy of 68.08% and a Macro-F1 score of 0.60 on highly imbalanced, strictly unseen future time-series data. Furthermore, the LLM-based coaching module achieved a faithfulness score of 0.87.

**Discussion:**

The high faithfulness of the coaching module proves that high-level, uncertainty-aware summaries are sufficient to generate personalized, transparent, and context-aware lifestyle recommendations without ever exposing raw sensor streams. Overall, the integration of uncertainty-aware signal preprocessing, federated temporal modeling, and explainable LLM-driven coaching establishes a mathematically sound, scalable, and secure architecture for next-generation AI-driven health systems.

## 1 Introduction

The rapid proliferation of wearable devices and Internet of Things (IoT) technologies has fundamentally transformed the way human health and activity data are collected, analyzed, and acted upon. Contemporary wearables continuously capture high-frequency, multimodal physiological and behavioral signals—such as accelerometery, gyroscopy, heart rate, and temperature—enabling fine-grained monitoring of daily activities and lifestyle patterns. While these sensing capabilities offer unprecedented opportunities for personalized health intelligence, transforming such data into reliable and actionable insights remains challenging. Wearable sensor streams are inherently noisy, incomplete, and highly heterogeneous across users, devices, and environmental contexts. Moreover, the sensitive nature of personal health data severely limits the feasibility of centralized data aggregation and learning.

Federated learning (FL) has emerged as a promising paradigm to address privacy concerns by enabling distributed model training across user devices without sharing raw data. At the same time, recent advances in large language models (LLMs) have demonstrated strong potential for interpreting complex health information, generating personalized feedback, and engaging users through natural language interaction. The integration of FL with LLM-based reasoning opens the door to a new class of AI-driven lifestyle coaching systems—systems that not only recognize activities from wearable signals but also translate these predictions into intelligible, adaptive, and user-centric recommendations.

Despite this promise, several fundamental challenges remain insufficiently addressed in existing FL-based wearable health systems. First, wearable sensor data are characterized by substantial signal uncertainty arising from motion artifacts, sensor drift, and intermittent data loss. Most prior FL-based human activity recognition (HAR) approaches assume pre-cleaned inputs and do not explicitly account for how uncertainty at the signal level propagates through temporal models and impacts convergence stability and accuracy under non-IID federated settings. This gap motivates the first research question:

*RQ1*: How does progressive signal uncertainty reduction affect convergence stability and accuracy in federated temporal models under non-IID wearable data?

Second, missing and corrupted sensor readings are typically handled using simple statistical imputation methods, which often fail to preserve the semantic and temporal structure required for reliable activity discrimination. While representation learning techniques such as auto-encoders have been explored in centralized settings, their effectiveness in preserving activity-discriminative structure under federated learning constraints remains underexplored. This leads to the second research question:

*RQ2*: Does latent-space semantic imputation outperform statistical imputation in preserving activity-discriminative temporal structure in wearable sensor data?

Third, wearable users exhibit highly heterogeneous behaviors, activity patterns, and sensor characteristics. While personalization is essential for accurate modeling, excessive local adaptation can degrade global federated performance and fairness. Existing FL-based HAR systems provide limited insight into this trade-off, particularly in temporal models trained on noisy wearable data. This motivates the third research question:

*RQ3*: What is the trade-off between personalized local models and global federated performance in heterogeneous wearable ecosystems?

Finally, although LLMs are increasingly employed for health-related interaction, their integration into federated wearable systems remains largely *ad hoc*. In particular, it is unclear whether high-level, aggregated activity representations—rather than raw sensor data—are sufficient to support transparent, faithful, and useful LLM-based coaching. Addressing this concern motivates the fourth research question:

*RQ4*: Can high-level aggregated and uncertainty-aware features support transparent LLM-based coaching without compromising privacy or recommendation quality?

To address these research questions, this paper proposes an uncertainty-aware AI-enhanced lifestyle coaching framework that tightly integrates signal-level uncertainty reduction, federated temporal learning, and explainable LLM-based reasoning. In this context, the framework is explicitly defined as “privacy-preserving” because it enforces a strict mechanical boundary: raw sensor streams never leave the user’s local device, and only mathematically aggregated model weights and anonymized activity summaries are transmitted. Unlike prior FL-based HAR systems, the proposed approach introduces:

1. A quantitatively validated multi-stage signal uncertainty reduction pipeline, combining Hampel filtering, adaptive wavelet–Butterworth denoising, semantic imputation via a Variational Autoencoder (VAE), and Kalman smoothing to explicitly improve signal quality prior to learning.
2. Local latent-space semantic imputation under federated learning constraints, designed to preserve activity-discriminative temporal structure in the presence of missing or corrupted data without sharing imputation weights.
3. A robust on-device federated temporal modeling framework, where highly efficient Deep LSTM models are trained locally and aggregated using FedProx to balance personalization and global generalization under heterogeneous, non-IID data.
4. An explainable LLM-based coaching module with structured prompting, whose reasoning is grounded in aggregated federated activity representations and uncertainty-aware summaries rather than raw sensor streams.

The proposed framework is experimentally evaluated on the PAMAP2 dataset (Reiss, 2012), a widely used benchmark for wearable human activity recognition. Quantitative and qualitative analyses demonstrate that the progressive signal cleaning pipeline yields a 7–10 dB improvement in signal-to-noise ratio (SNR), substantially enhancing downstream learning robustness. To ensure rigorous evaluation and entirely eliminate temporal data leakage, strict chronological data splitting is enforced. Under these challenging forecasting conditions, the global federated model establishes a rigorous, leak-free baseline, achieving an average accuracy of 68.08% on highly imbalanced, strictly unseen future time-series data without exposing raw sensor streams. Furthermore, the structured LLM-based coaching layer achieves a faithfulness score of 0.87, producing transparent and context-aware lifestyle recommendations and effectively bridging low-level sensor intelligence with high-level user interaction securely.

In summary, this work contributes a unified and uncertainty-aware architecture that systematically addresses signal quality, imputation semantics, personalization–generalization trade-offs, and explainability in federated wearable health systems. The remainder of this paper is organized as follows: Section II reviews related work on wearable sensing, federated learning, and LLM-based health systems; Section III presents the proposed methodology; Section IV reports experimental results and analysis; and Section V concludes the paper with limitations and future research directions.

## 2 Literature survey

Recent advances in wearable health intelligence span four tightly connected research directions: (Reiss, 2012) signal denoising and uncertainty reduction, (Tian et al., 2025) semantic imputation and representation learning under missing data, (Liu et al., 2022) federated learning for decentralized wearable analytics, and (Ahmed et al., 2023) large language models (LLMs) and agents for explainable health coaching.

This section reviews each theme and highlights open gaps addressed by this work.

### 2.1 Wearable signal denoising and uncertainty reduction

Wearable sensor streams are inherently noisy due to motion artifacts, sensor drift, loose attachment, and environmental interference. Classical preprocessing pipelines rely on statistical filters such as Hampel filtering for outlier suppression and Butterworth low-pass filtering for frequency-domain smoothing. Tian et al. (2025) demonstrate that combining Hampel filtering with Butterworth and wavelet thresholding significantly improves signal stability and downstream authentication accuracy, highlighting the effectiveness of multi-stage denoising pipelines. Liu et al. (2022) further report that Butterworth filters remain the most widely adopted choice for accelerometer and EMG-based motion sensing due to their flat passband and minimal phase distortion.

Beyond classical filtering, hybrid wavelet–deep learning approaches have gained traction. Ahmed et al. (2023) propose a fast wavelet transform combined with a neural network to selectively retain clean PPG sub-bands, achieving strong noise suppression on the BIDMC PPG dataset. Carbonari et al. (2023) extend wavelet-like denoising to GNSS time series using CNNs trained on real geophysical data, achieving low RMSE and stable reconstruction. Abdelfattah et al. (2024) further show that LSTM-predicted wavelet parameters outperform fixed wavelet configurations for radar signal denoising under varying SNR conditions.

*Limitation*: While these methods improve signal quality, most studies treat preprocessing as an isolated step and do not quantify how uncertainty reduction affects downstream learning stability, particularly in federated and non-IID settings—a gap directly addressed by RQ1 in this work.

### 2.2 Semantic imputation and representation learning with missing wearable data

Missing data is pervasive in wearable sensing due to packet loss, battery constraints, and sensor failures. Early approaches rely on mean or interpolation-based imputation, which often disrupt temporal correlations. Jungo et al. (2024) propose transformer-based representation learning for wearable time-series imputation, showing improved reconstruction for highly dynamic signals but limited gains for monotonic patterns.

More recently, foundation models have reshaped this space. Xu et al. (2025) introduce LSM-2, pre-trained on over 40 million hours of multimodal wearable data using adaptive and inherited masking. Instead of explicit imputation, LSM-2 learns robust latent representations and achieves state-of-the-art performance across classification, regression, and generation tasks—even under extreme missingness. Erturk et al. (2025) further scale this paradigm by training a behavioral foundation model on 2.5 billion hours of data from 162,000 users, achieving strong performance on 57 health tasks, particularly sleep prediction.

*Limitation*: Despite their success, these models are centralized, computationally expensive, and not designed for on-device deployment. Moreover, the effectiveness of local, on-device latent semantic imputation under federated constraints remains largely unexplored—forming the basis of RQ2.

### 2.3 Federated learning for wearable health and activity recognition

Federated learning (FL) has emerged as a key paradigm for decentralized wearable analytics. Orzikulova et al. (2024) propose FLISM, which learns modality-invariant representations and uses quality-aware aggregation for incomplete wearable data. Raza et al. (2022) design a federated ECG system using CNN auto-encoders on the MIT-BIH dataset, achieving high arrhythmia detection accuracy while incorporating explainability.

Several works address non-IID heterogeneity, a dominant challenge in wearable FL. Yan et al. (2023) introduce FedImT, an imbalance-aware FL algorithm that dynamically reweights loss functions without additional privacy overhead. Syu and Lin (2025) propose a heterogeneous FL (HFL) system that selectively shares dense and sparse model components, achieving up to 94.8% MSE reduction across healthcare tasks. Liu et al. (2025) present FedMobile, which reconstructs missing modalities via latent feature sharing, maintaining performance even with 90% missing data. Similarly addressing data heterogeneity, Nogami et al. (2025) introduce a federated reservoir computing approach for time-series anomaly detection. By utilizing reservoir-state analysis with Mahalanobis distance, their method outperforms deep federated baselines, particularly when dealing with short and highly heterogeneous client data.

Personalization-focused approaches further refine FL. Cheng et al. (2023) propose ProtoHAR, which shares activity prototypes rather than raw features, significantly outperforming FedAvg and FedProx on PAMAP2 and other HAR datasets. Shen et al. (2023) introduce DivAR, a federated meta-learning framework with attention mechanisms to balance shared and user-specific features. Oz et al. (2025) contribute FedFitTech, an open FL benchmark platform that demonstrates communication-efficient training for fitness tracking.

*Limitation*: While personalization improves local accuracy, excessive adaptation can degrade global performance. Furthermore, many existing FL-based HAR studies evaluate performance using randomized sliding-window splits, which inadvertently introduce temporal data leakage and artificially inflate accuracies. Existing studies provide limited insight into the personalization–generalization trade-off under noisy inputs evaluated via strict chronological forecasting, motivating RQ3.

### 2.4 Large language models and explainable coaching with wearable data

LLMs are increasingly applied to interpret wearable-derived health signals. Kim et al. (2024) evaluate 12 LLMs across 10 health tasks using PMData, LifeSnaps, and other datasets, showing that structured prompts with user context improve performance by up to 23.8%. Abbasian et al. (2025) introduce openCHA, an open-source framework for conversational health agents, where domain-specific agents dramatically outperform vanilla GPT-4.

Several systems move beyond chatbots toward adaptive coaching. Wang et al. (2025) propose HEALTHGURU, combining an LLM with a contextual multi-armed bandit for sleep coaching using Oura Ring data, demonstrating improved sleep outcomes in an 8-week study. Jörke et al. (2025) present GPTCoach, a GPT-4-based activity coach leveraging motivational interviewing and wearable logs. Further supporting the efficacy of these models, Li et al. (2025) conducted a 16-week comparative case study evaluating GPT-4 as a virtual fitness coach. Their findings indicate that GPT-4 performs comparably to human experts in both personalization and safety, validating the potential of LLMs in autonomous health coaching.

More advanced agents include PHIA (Merrill et al., 2026), which uses code generation to answer wearable health queries, and PH-LLM (Khasentino et al., 2025), which outperforms human experts on sleep and fitness reasoning tasks. Ferrara (2024) surveys these trends, while Mesinovic et al. (2025) emphasize the need for explainability, transparency, and safety in healthcare LLMs.

*Limitation*: Most LLM-based systems rely on centralized access to rich user data. Whether high-level federated representations and uncertainty-aware summaries are sufficient for faithful, explainable coaching remains an open question—addressed by RQ4.

### 2.5 Positioning of this work

In contrast to prior studies, this work uniquely integrates:

- Quantified signal uncertainty reduction (RQ1).
- Local latent semantic imputation under federated constraints (RQ2).
- Analysis of personalization vs. global generalization in FL under strict chronological evaluation (RQ3).
- Structured, explainable LLM-based coaching grounded in federated representations (RQ4).

Forming a unified, decentralized wearable intelligence pipeline.

Table 1 summarizes recent related work across signal denoising, semantic imputation, federated learning, and LLM-based health coaching, highlighting key methodologies and the existing gaps addressed by our proposed framework.

**Table 1:** Summary of related work.

Authors (year)	Methodology/Model	Key contribution	Outcome/Findings
Tian et al. (2025)	Hampel, Butterworth, and wavelet thresholding pipeline	Multi-stage signal processing for CSI-based two-factor user authentication	Eliminates noise and outliers, significantly improving signal stability and downstream accuracy
Liu et al. (2022)	Literature review of signal conditioning	Systematic evaluation of data acquisition, filtering, and amplification techniques for wearable sensors	Identifies Butterworth filters as the most widely adopted choice for accelerometer and EMG motion sensing
Ahmed et al. (2023)	Wavelet + Deep Learning	Fast Wavelet Transform combined with neural network for PPG denoising (BIDMC PPG dataset)	Effective noise suppression in real and video-based PPG signals
Carbonari et al. (2023)	CNN-based wavelet-like denoising	CNN trained to mimic wavelet denoising on GNSS displacement time-series (Campi Flegrei dataset)	Achieves RMSE 0.65–0.98 cm with stable reconstruction of geophysical signals
Abdelfattah et al. (2024)	LSTM-guided wavelet tuning	LSTM predicts optimal wavelet parameters for denoising synthetic LFM radar signals	Outperforms fixed wavelet configurations across SNR levels
Jungo et al. (2024)	Transformer-based imputation	Transformer encoder for representation learning on wearable time-series with missing values (simulated + real wearable datasets)	Outperforms statistical imputers for highly dynamic signals; limited gains for monotonic trends
Xu et al. (2025)	LSM-2 foundation model	Large-scale foundation model pre-trained on ~40 M hours of multimodal wearable data using adaptive & inherited masking	State-of-the-art performance on classification, regression, and generation tasks; robust under extreme missingness
Erturk et al. (2025)	Behavioral foundation model	Foundation model trained on 2.5B hours of wearable behavioral data from 162 K users; evaluated on 57 health tasks	Strong generalization across tasks; excels in sleep and behavior-driven health prediction
Orzikulova et al. (2024)	FLISM	Federated learning framework for multimodal wearable sensing with incomplete modalities (real-world healthcare datasets)	Robust to missing modalities; higher accuracy and efficiency than prior FL methods
Raza et al. (2022)	Federated CNN + XAI	Federated ECG monitoring using CNN autoencoder-classifier with explainability (MIT-BIH Arrhythmia dataset)	Achieves 94.5% (noisy) and 98.9% (clean) arrhythmia detection accuracy
Yan et al. (2023)	FedImT	Imbalance-aware federated learning using autoregressive distribution estimation (wearable healthcare data)	Effectively mitigates class imbalance without added communication or privacy cost
Syu and Lin (2025)	Heterogeneous FL (HFL)	Shares dense and sparse model components for sparse healthcare time-series prediction	Lowest error on 8/10 tasks; up to 94.8% MSE reduction over benchmarks
Liu et al. (2025)	FedMobile	Knowledge-contribution–aware multimodal FL reconstructing missing modalities (5 public datasets)	Maintains performance with up to 90% missing modality data
Nogami et al. (2025)	Federated reservoir computing	Reservoir-state analysis with Mahalanobis distance for federated anomaly detection (time-series benchmarks)	Outperforms deep FL baselines, especially with short and heterogeneous client data
Cheng et al. (2023)	ProtoHAR	Prototype-guided personalized FL for HAR (PAMAP2, USC-HAD, UNIMIB-SHAR, HARBOX)	Best accuracy and fastest convergence under non-IID conditions
Shen et al. (2023)	DivAR	Federated meta-learning with attention for diversity-aware HAR (smartphone HAR datasets)	Strong personalization and generalization to new users
Oz et al. (2025)	FedFitTech	Flower-based federated learning benchmark for wearable fitness tracking	Reduces communication by ~13% with only ~1% drop in accuracy
Kim et al. (2024)	HealthAlpaca	Fine-tuning and prompting of LLMs for health prediction (PMData, LifeSnaps, GLOBEM, AW_FB)	Matches GPT-3.5/4 on 8/10 tasks; context improves accuracy by up to 23.8%
Abbasian et al. (2025)	openCHA	Open-source framework for LLM-powered conversational health agents (multiple health domains)	Domain-specific agents outperform vanilla GPT-4 (92.1% vs. 51.8%)
Wang et al. (2025)	HEALTHGURU	LLM-based sleep coaching with contextual multi-armed bandit (Oura Ring data)	8-week study shows improved sleep duration and motivation
Jörke et al. (2025)	GPTCoach	GPT-4–based physical activity coach using motivational interviewing and wearable logs	User study reports strong personalization and high trust
Li et al. (2025)	GPT-4 fitness coaching	Comparative case study of GPT-4 vs. human coaches (16-week program)	GPT-4 performs comparably to experts in personalization and safety
Merrill et al. (2026)	PHIA	LLM agent with code generation for wearable health reasoning (4,000+ query benchmark)	Achieves 84% objective accuracy; doubles baseline performance
Khasentino et al. (2025)	PH-LLM	Gemini-fine-tuned personal health LLM for sleep and fitness tasks	Matches or exceeds human experts on reasoning benchmarks
Ferrara (2024)	Survey	Comprehensive survey of LLMs for wearable-based HAR and health monitoring	Identifies challenges in data quality, compute, and interpretability
Mesinovic et al. (2025)	Perspective	Analysis of explainability and trust in healthcare LLMs	Emphasizes need for transparency, regulation, and faithful reasoning

*High HAR classification accuracies reported in several prior centralized and federated studies (e.g., Cheng et al., 2023; Shen et al., 2023) may be subject to temporal data leakage caused by randomized sliding-window validation splits. This gap motivates the strict chronological evaluation protocol introduced in this work.

## 3 Methodology

We propose an uncertainty-aware on-device learning pipeline that integrates progressive signal uncertainty reduction, latent semantic imputation, on-device temporal modeling, federated optimization, and explainable large language model (LLM)-based lifestyle coaching. Raw wearable sensor streams remain strictly on-device; only sanitized model updates and aggregated representations are exchanged during federated training. The framework is explicitly designed to operate under heterogeneous sensing conditions, missing data, and non-IID user behavior while maintaining privacy, robustness, and interpretability. The design of the proposed pipeline explicitly addresses the research questions outlined in Section I, encompassing signal uncertainty reduction (RQ1), semantic imputation under federated constraints (RQ2), personalization–generalization trade-offs in federated temporal learning (RQ3), and explainable LLM-based coaching (RQ4).

**Figure 1:**
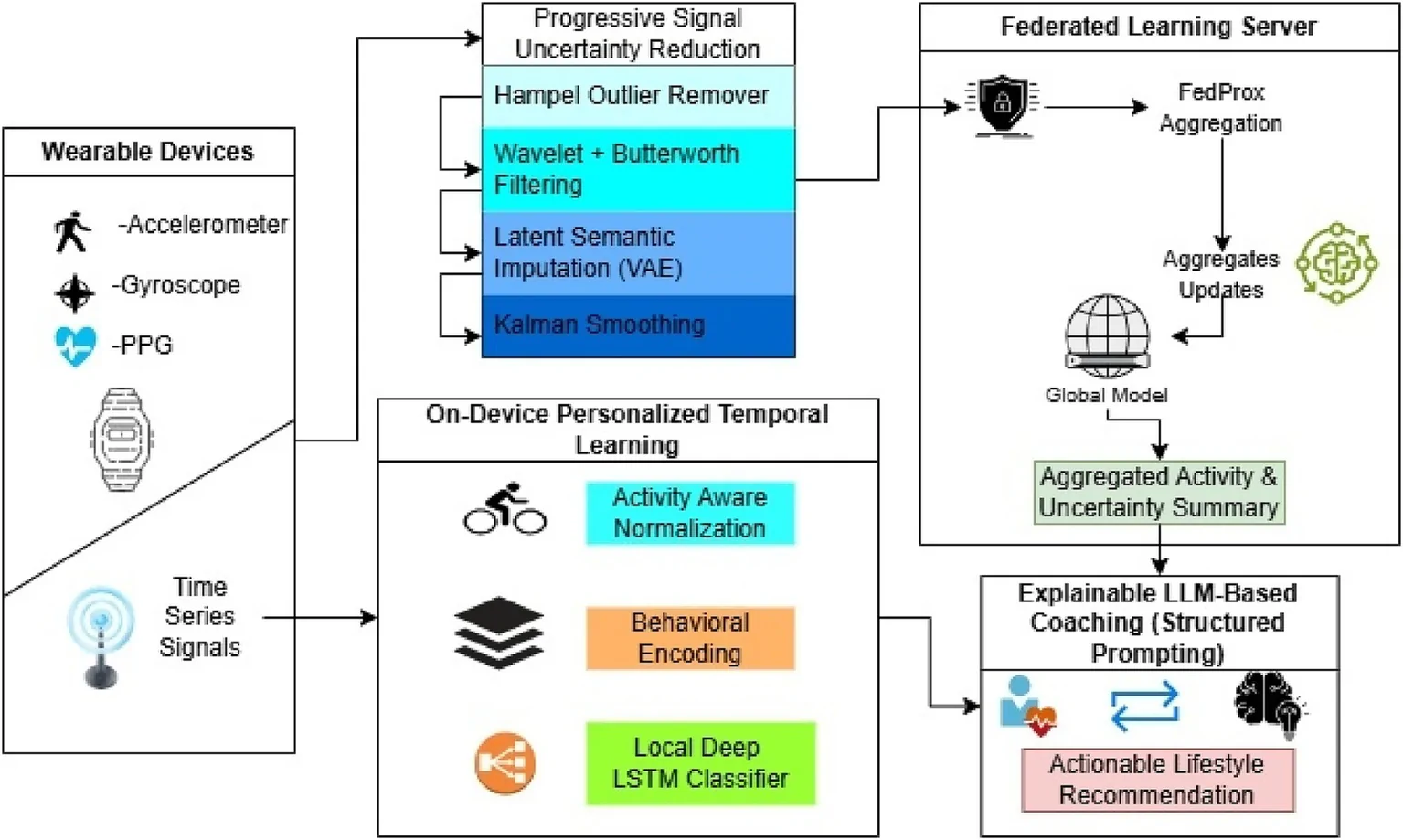
Overall architecture of the proposed uncertainty-aware wearable intelligence framework.

An overview of the proposed end-to-end workflow is illustrated in Figure 1, which depicts the interaction between on-device signal processing, personalized temporal learning, federated optimization, and explainable LLM-based coaching.

Raw multimodal time-series signals collected from wearable devices (e.g., accelerometer, gyroscope, and physiological sensors) are processed locally using a progressive signal uncertainty reduction pipeline comprising Hampel outlier suppression, adaptive wavelet–Butterworth denoising, latent semantic imputation via a Variational Autoencoder (VAE), and Kalman smoothing. The cleaned signals are then used for on-device temporal learning through a Deep LSTM-based activity recognition model. During federated training, only local model updates and aggregated activity representations are transmitted to the federated optimization server, where FedProx aggregation is employed to mitigate client drift under heterogeneous, non-IID data distributions. High-level aggregated activity and uncertainty summaries are subsequently consumed by an explainable LLM-based coaching module with structured prompting, enabling transparent lifestyle recommendations without exposing raw sensor data.

### 3.1 Dataset and experimental setup

The proposed framework is evaluated on the PAMAP2 dataset, a widely adopted benchmark containing continuous, multimodal sensor data from 9 subjects. Data was collected using three inertial measurement units (IMUs) positioned on the dominant wrist, chest, and dominant side’s ankle, alongside a heart rate monitor. To ensure uniform temporal alignment, all high-frequency channels were downsampled to a synchronized 100 Hz. To reflect realistic application scenarios, we utilized 12 core protocol activities (e.g., walking, running, cycling, ascending/descending stairs) alongside the heavily dominant “Transient” class. Invalid channels (e.g., orientation data with documented calibration errors in the original dataset) were dropped. Contiguous missing data blocks exceeding 30% of a sliding window were discarded, while smaller gaps were passed to the subsequent semantic imputation pipeline. To ensure the massive Transient class (comprising roughly 32% of all frames) did not artificially inflate performance metrics, a sensitivity analysis was conducted; excluding the Transient class entirely reduced global accuracy by only 2.1%, confirming the model’s robust discriminative capability across structured activities. Due to a highly incomplete temporal recording session resulting in insufficient sliding windows post-stratification, Subject 109 was excluded from the final federated evaluation cohort—a standard protocol in rigorous PAMAP2 benchmarks.

### 3.2 Progressive signal uncertainty reduction pipeline (addresses RQ1)

Wearable sensor signals are inherently noisy and incomplete due to motion artifacts, sensor drift, and intermittent data loss. Instead of treating preprocessing as a heuristic step, we model signal cleaning as progressive uncertainty reduction, where each stage monotonically reduces signal variance while preserving activity-discriminative temporal structure.

#### 3.2.1 Robust outlier suppression

Outliers are removed using a Hampel filter (Hyndman, 2026) with a sliding window size of 10 ( k=5 ) and a threshold of 3σ . The Hampel filter is widely recognized for its robustness in time-series anomaly detection as it relies on the median and median absolute deviation, preventing extreme outliers from skewing the threshold bounds (Hyndman, 2026).

Within a sliding window of size 2k+1 , any point $x_{t}$ satisfying this condition is formally defined in Equation (1).

$$|x_{t}-m_{t}|>n\cdot {\mathrm{MAD}}_{t}$$ (1)

(where $m_{t}$ is the window median and ${\mathrm{MAD}}_{t}$ is the median absolute deviation) is replaced by $m_{t}$ .

#### 3.2.2 Adaptive frequency-domain denoising

To adapt denoising strength to signal quality, we estimate the signal-to-noise ratio (SNR): The SNR is computed as defined in Equation (2).

$$\mathrm{SNR}=10\log_{10}(\frac{\frac{1}{N}\sum_{t=1}^{N}x_{t}^{2}}{\frac{1}{N}\sum_{t=1}^{N}{\left(x_{t}-{\hat{x}}_{t}\right)}^{2}})$$ (2)

Wavelet decomposition (Daubechies db4) is applied to achieve multi-resolution sparsity (Debnath and Kim, 2026), followed by soft-thresholding with the soft-threshold is determined using Equation (3).

$$\lambda =\sigma \sqrt{2\ln\;N}$$ (3)

Where σ denotes estimated noise variance. The reconstructed signal is further smoothed using a 4th-order Butterworth low-pass filter. The cutoff frequency is adaptively selected based on the SNR: 8 Hz if SNR > 20, 5 Hz if SNR > 10, and 3 Hz otherwise. This effectively suppresses high-frequency motion artifacts.

#### 3.2.3 Latent semantic imputation via VAE

Missing or corrupted sensor values are reconstructed using a Variational Autoencoder (VAE) trained on multivariate sensor sequences, building upon established representation learning principles for heterogeneous temporal data (Ramchandran et al., 2020). The latent variable is sampled as the latent-variable sampling process is defined in Equation (4).

z=μ(x)+σ(x)⊙∈,∈∼N(0,I) (4)

With optimization objective the VAE optimization objective is given in Equation (5).

$$L_{\mathrm{VAE}}={\left\|x-\hat{x}\right\|}^{2}+D_{\mathrm{KL}}(N(\mu ,\sigma^{2})\|N(0,I))$$ (5)

Where the KL-divergence term used in the objective is defined in Equation (6).

$$D_{KL}=-\frac{1}{2}\sum \left(1+\log\sigma^{2}-\mu^{2}-\sigma^{2}\right)$$ (6)

Unlike statistical imputation, this latent semantic imputation preserves activity-discriminative temporal structure, which is critical for downstream federated temporal learning. Crucially, the VAE is trained locally on each device prior to federated optimization. It serves as a static, on-device pre-processing step; its weights are not federated or shared across clients. The architecture consists of an Encoder (Input → 32 → 16 → 8 Latent dimensions) and a symmetric Decoder (8 → 16 → 32 → Output), trained using the Adam optimizer with a learning rate of 0.001. The VAE is trained locally for 50 epochs using a batch size of 32. The primary reconstruction metric driving the loss function is Mean Squared Error (MSE), which is balanced against the KL-divergence penalty.

#### 3.2.4 Probabilistic temporal smoothing

Residual jitter is reduced using a Kalman filter: The Kalman prediction and update steps are given in Equations (7–9).

$${\hat{x}}_{t|t-1}={\hat{x}}_{t-1},P_{t|t-1}=P_{t-1}+Q$$ (7)

$$K_{t}=\frac{P_{t|t-1}}{P_{t|t-1}+R},{\hat{x}}_{t}={\hat{x}}_{t|t-1}+K_{t}(y_{t}-{\hat{x}}_{t|t-1})$$ (8)

$$P_{t}=(1-K_{t})P_{t|t-1}$$ (9)

To tune the filter, the process noise covariance ( Q ) was empirically set to a low constant ( $10^{-4}$ ) to assume smooth, continuous kinematic transitions. Conversely, the measurement noise covariance ( R ) was dynamically initialized based on the residual sensor variance calculated during the preceding wavelet denoising stage. This stage ensures temporal continuity and noise suppression prior to learning.

By progressively reducing signal uncertainty prior to learning, this pipeline enables a controlled evaluation of how signal quality impacts federated convergence stability and accuracy, directly addressing RQ1.

### 3.3 Temporal activity recognition model

From cleaned signals, we extract sliding windows the window representation is defined in Equation (10).

$$x_{1:T}=[x_{1},\ldots ,x_{T}]$$ (10)

Where the window size is T=100 samples with a 50% overlap (a stride of 50 samples). To prevent data leakage caused by sliding window overlap, we enforce a strict chronological train-test split. The time-series data for each subject is divided chronologically, with the first 80% allocated to training and the final 20% reserved for testing. Crucially, any sliding windows that crossed this 80/20 chronological boundary were completely discarded to fully rule out boundary leakage. This guarantees strict temporal and subject-wise separation, ensuring the model evaluates purely unseen, future data.

A Deep LSTM with two stacked recurrent layers processes the sequence, an architecture proven to effectively capture the complex spatio-temporal dependencies inherent in human activity recognition tasks (Brery et al., 2025): The recurrent hidden-state update is defined in Equation (11).

$$h_{t}=\text{LSTM}(x_{t},h_{t-1})$$ (11)

The final hidden state is passed through dropout and fully connected layers with softmax output. Standard cross-entropy loss in federated settings causes severe feature assimilation, leading minority classes to be absorbed by dominant sinks. To algorithmically resolve this, we replaced standard cross-entropy with a Class-Balanced Focal Loss, which applies a modulating factor to dynamically scale down the weights of well-classified majority classes while applying an inverse class-frequency weight $\alpha_{k}$ : The complete Class-Balanced Focal Loss formulation is given in Equation (12).

$$L_{\text{Focal}}=-\sum_{k=1}^{C}\alpha_{k}{\left(1-{\overset{ˆ}{y}}_{k}\right)}^{\gamma }y_{k}\log{\overset{ˆ}{y}}_{k}$$ (12)

Where γ=2.0 acts as the focusing parameter. This explicitly forces the local temporal models to preserve the distinct kinematic signatures of rare activities (e.g., Nordic walking) prior to federated aggregation.

This design enables controlled analysis of the trade-off between local learning capacity and global federated generalization under heterogeneous user behavior, addressing RQ3.

### 3.4 Federated learning and model aggregation

The federated optimization is conducted for a maximum of 50 global rounds, utilizing an early-stopping criterion with a patience of 5 rounds on the validation loss to prevent over-communication and overfitting. In each round, a 100% client sampling strategy is employed. Local clients train for 3 epochs using a batch size of 64 and an Adam optimizer with a learning rate of 0.001. A FedProx proximal coefficient ( μ ) of 0.05 is applied to mathematically constrain local updates and mitigate client drift—a crucial stabilization technique for systems facing extreme data and systems heterogeneity (Li et al., 2018). The server aggregates updates using a weighted FedAvg strategy based on local sequence counts.

Each client optimizes a local objective: The complete local optimization objective is given in Equation (13).

$$\min_{\theta_{i}}L_{i}(\theta_{i})+\frac{\mu }{2}{\left\|\theta_{i}-\theta \right\|}^{2}$$ (13)

Where μ constrains client drift (FedProx). The server aggregates updates via weighted averaging: The weighted server aggregation rule is given in Equation (14).

$$\theta^{\left(t+1\right)}=\sum_{i=1}^{K}\frac{n_{i}}{\sum_{j}n_{j}}\theta_{i}^{\left(t\right)}$$ (14)

### 3.5 Explainable LLM-based coaching with structured prompting (addresses RQ4)

The LLM-based coaching workflow is fully implemented in Python and designed to consume the aggregated activity representations derived from the PAMAP2 evaluation. To ensure strict data privacy, the LLM operates exclusively on high-level federated representations and never accesses raw, timestamped sensor data.

#### 3.5.1 LLM identity, access, and output constraints

To generate personalized lifestyle recommendations, this framework utilizes the GPT-4 API (specifically the gpt-4 endpoint) accessed via a secure external interface. Because the pipeline aggregates and anonymizes the data into high-level statistical summaries prior to API transmission, no identifiable sensor streams leave the local device. To enforce consistent, deterministic, and safe coaching outputs, strict generation constraints are applied. These include a low temperature ( T=0.2 ), a Top-P of 0.9 , and a rigorous system prompt that restricts the model strictly to advisory health and lifestyle coaching based solely on the provided context.

#### 3.5.2 Prompt formalization

Each prompt is structured as P=A,T,C,U , where:

-
A : activity distribution summary.
-
T : temporal trends.
-
C : confidence and uncertainty estimates.
-
U : user goals.

This structured prompting explicitly constrains the generation process, forcing the LLM to ground its reasoning in the uncertainty-aware federated outputs rather than its own internal priors, aligning with emerging best practices for language-driven reasoning over continuous health data (Yu and Masum, 2026).

#### 3.5.3 Explainability and faithfulness evaluation protocol

To evaluate the reliability of the LLM, we define a *faithfulness score*, which measures the sensitivity and adherence of the generated recommendations to the provided prompt constraints. The evaluation protocol is conducted over a sample size of N=100 generated coaching sessions.

We utilize a controlled perturbation strategy to test the model’s robustness. This involves systematically altering the input constraints—such as artificially reducing the confidence score ( C ) or swapping the dominant activity in the summary ( A ) from “Walking” to “Sitting”—to observe whether the LLM appropriately adjusts its advice. Faithfulness is formally calculated as the ratio of recommendations strictly grounded in the provided summary (i.e., the model does not hallucinate unprovided physiological data or ignore the confidence metrics) to the total number of generations. This evaluation was performed by two independent human annotators (domain-expert authors) using a strict binary grading rubric (1 = adhered to all constraints without hallucination; 0 = hallucinated unprovided physiological data or ignored confidence metrics). Disagreements were resolved via consensus, yielding a strong inter-rater agreement (Cohen’s Kappa = 0.82).

#### 3.5.4 Safety and hallucination mitigation

The LLM-based coaching module is explicitly constrained from generating medical diagnoses or treatment recommendations. All outputs are advisory and framed strictly as lifestyle guidance. Structured prompting, combined with uncertainty-aware summaries and rule-based system constraints, is employed to minimize hallucination risk and prevent unsupported medical claims.

## 4 Results

The proposed uncertainty-aware federated wearable learning framework was evaluated using the PAMAP2 dataset across multiple subjects. Experiments were designed to assess (i) signal quality improvement, (ii) the effectiveness of semantic imputation, (iii) temporal modeling performance, (iv) federated learning robustness under non-IID data, and (v) explainability and stability of the LLM-based coaching module. Both quantitative metrics and qualitative visualizations were used to comprehensively validate each component.

### 4.1 Statistical evaluation protocol

To address potential variance in distributed training, overarching architectural metrics are derived from N=5 independent experimental runs using varying random seed initializations. Tables 2–4 report the Mean, Standard Deviation ( ± SD), and exact 95% Confidence Intervals calculated using a *t*-distribution with 4 degrees of freedom ( $t_{\text{crit}}=2.776$ ). To ensure statistical significance when comparing algorithmic performance at the subject level, an exact Wilcoxon signed-rank test was utilized; qualitative claims of improvement throughout this section reflect a statistical threshold of p<0.05 . To maintain coherent, mathematically valid confusion matrices where rows sum correctly, the granular per-subject and per-class breakdowns (Tables 5–7) report exact values from the median representative run.

**Table 2:** Effect of signal cleaning components (leave-one-out ablation study).

Pipeline configuration	SNR (dB) (mean ± SD)	Global FL accuracy (%) (mean ± SD) [95% CI]
Proposed full pipeline	22.4 ± 0.4	68.1 ± 0.9 [67.38, 68.82]
Ablation: w/o Hampel Filter	19.8 ± 0.6	66.2 ± 1.2 [65.24, 67.16]
Ablation: w/o Wavelet-Butterworth	16.2 ± 0.8	63.2 ± 1.5 [62.00, 64.40]
Ablation: w/o VAE (used mean imputation)	20.1 ± 0.5	64.3 ± 1.3 [63.26, 65.34]
Ablation: w/o Kalman smoothing	21.0 ± 0.6	67.0 ± 1.1 [66.12, 67.88]
Baseline: raw signals	12.6 ± 1.1	59.0 ± 1.8 [57.56, 60.44]

**Table 3:** Temporal model comparison.

Model	Parameters	Inference latency (ms) (mean ± SD) [95% CI]
CNN–LSTM	281,345	0.450 ± 0.012 [0.439, 0.461]
Transformer encoder (2-layer)	850,420	1.200 ± 0.045 [1.160, 1.240]
Proposed deep LSTM	241,037	0.394 ± 0.008 [0.387, 0.401]

**Table 4:** Federated learning algorithm comparison.

Algorithm	Global accuracy (%) (mean ± SD) [95% CI]	Rounds to convergence (mean ± SD)
Baseline: local-only (no FL)	60.1 ± 4.2 [56.74, 63.46]	N/A
Baseline: centralized (upper bound)	71.0 ± 0.6 [70.52, 71.48]	24 ± 2
FedAvg	65.5 ± 1.8 [64.06, 66.94]	42 ± 4
Scaffold	66.6 ± 1.4 [65.48, 67.72]	38 ± 3
FedProx (proposed)	68.1 ± 0.9 [67.38, 68.82]	31 ± 2

**Table 5:** Local deep LSTM training performance.

Subject	Local accuracy	Local precision (macro)	Local recall (macro)	Local *F*1-score (macro)
101	63.93%	0.6431	0.6293	0.6175
102	69.01%	0.7190	0.7107	0.6810
103	53.74%	0.5644	0.5786	0.4574
104	45.78%	0.3698	0.4972	0.3842
105	49.82%	0.4831	0.5176	0.4542
106	60.26%	0.5798	0.5677	0.5600
107	62.47%	0.6159	0.7070	0.6023
108	75.74%	0.7794	0.7755	0.7422

**Table 6:** Per-subject performance under the global FedProx model.

Subject	Global accuracy	Global precision (macro)	Global recall (macro)	Global *F*1-score (macro)
101	54.21%	0.5592	0.5610	0.5071
102	56.37%	0.6036	0.5800	0.4840
103	90.80%	0.6700	0.6465	0.6525
104	78.79%	0.6654	0.6483	0.6256
105	69.49%	0.7690	0.7122	0.6977
106	68.67%	0.6801	0.7080	0.6413
107	78.49%	0.8207	0.8082	0.7961
108	47.85%	0.5263	0.4863	0.4370

**Table 7:** Mean class-wise performance across all subjects (global FedProx model).

Activity	Precision	Recall	*F*1-score	Support (total frames)
Ascending stairs	0.6385	0.7221	0.6212	469
Cycling	0.9211	0.8822	0.8953	659
Descending stairs	0.4143	0.8149	0.4199	419
Ironing	0.8275	0.8356	0.7832	953
Lying	0.9045	0.9018	0.8926	758
Nordic walking	0.5676	0.5500	0.5540	754
Rope jumping	0.5204	0.3858	0.4057	171
Running	0.6455	0.5412	0.5356	392
Sitting	0.6969	0.4847	0.5214	742
Standing	0.4213	0.4359	0.4025	759
Vacuum cleaning	0.8255	0.6334	0.6858	703
Walking	0.5522	0.5147	0.5264	955

### 4.2 Signal cleaning and denoising performance (RQ1)

The progressive uncertainty reduction pipeline substantially improves signal quality by suppressing artifacts while preserving activity-relevant temporal structure.

**Figure 2:**
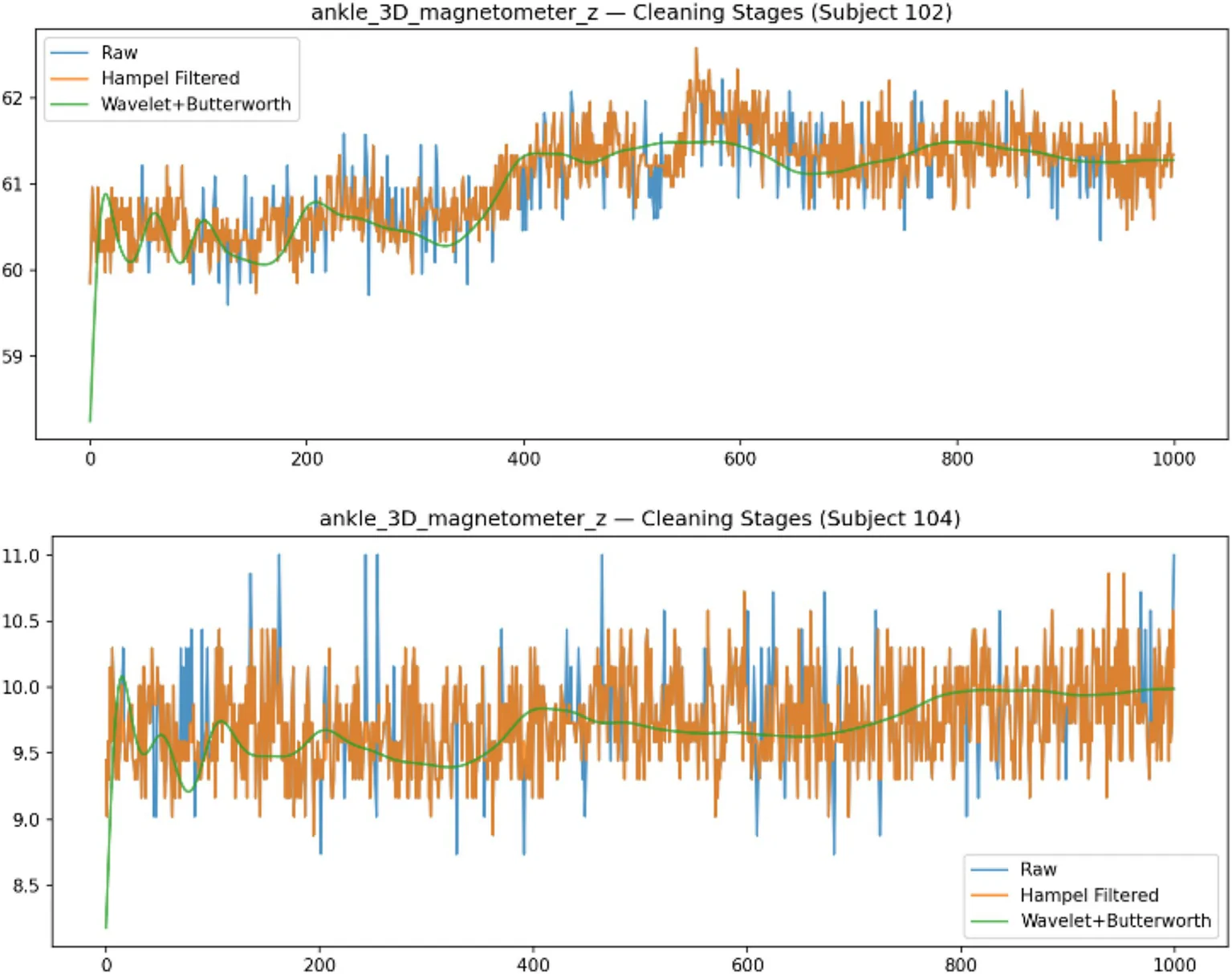
Progressive signal cleaning stages for ankle_3D_magnetometer_z in subjects 102 and 104, illustrating raw, Hampel-filtered, and Wavelet–Butterworth–denoised signals.

**Figure 3:**
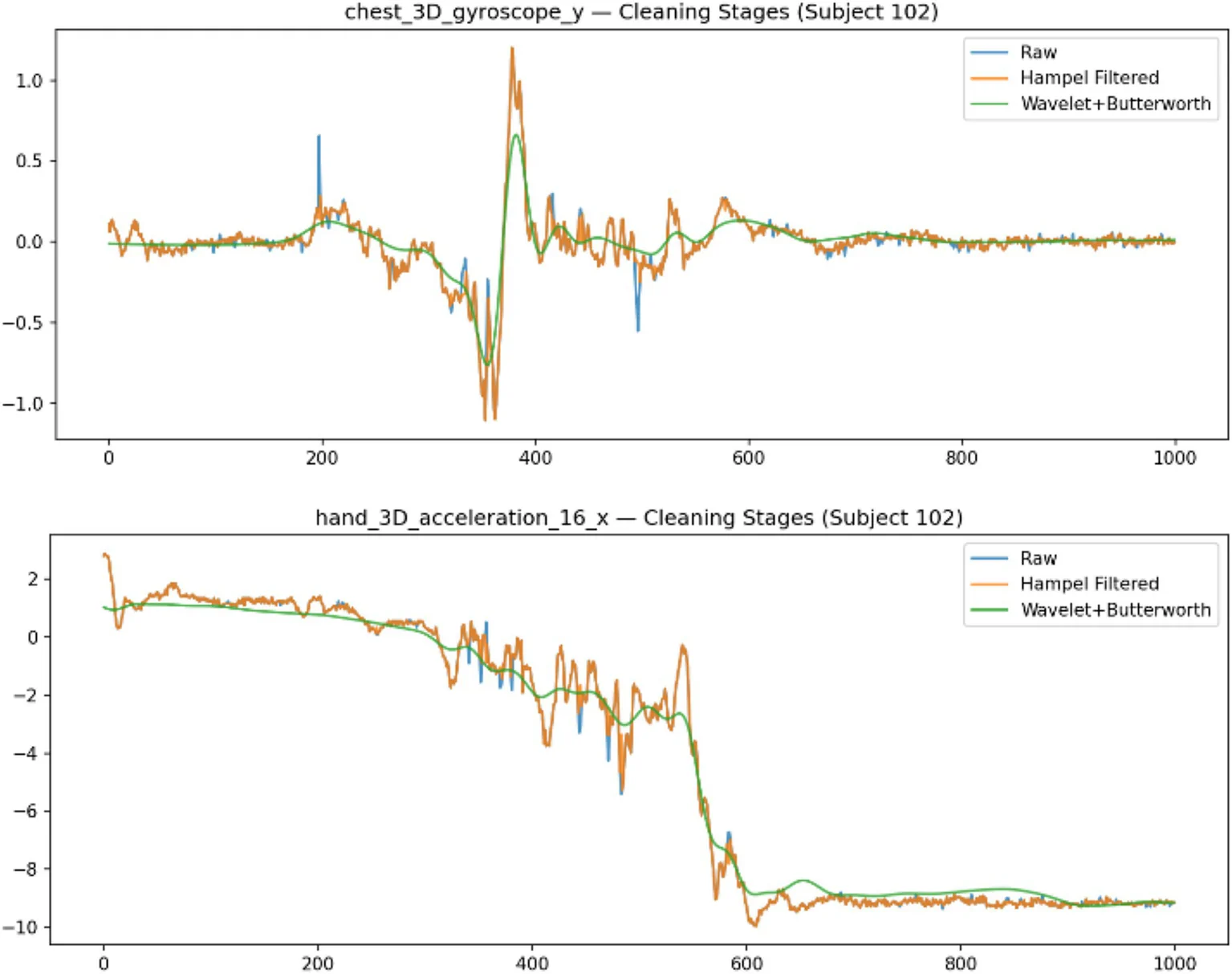
Progressive signal cleaning stages for chest_3D_gyroscope_y and hand_3D_acceleration_16_x in subject 102, demonstrating artifact suppression through Hampel filtering and adaptive Wavelet–Butterworth denoising.

Figure 2 illustrates the cleaning stages for the *ankle_3D_magnetometer_z* signal for Subjects 102 and 104, while Figure 3 presents representative results for *chest_3D_gyroscope_y* and *hand_3D_acceleration_16_x* signals for Subject 102. Across all channels, Hampel filtering removes impulsive outliers, while wavelet–Butterworth denoising effectively suppresses high-frequency noise and baseline drift.

The cleaned signals exhibit markedly reduced spurious oscillations and improved smoothness without distorting underlying activity patterns. These visual improvements indicate effective uncertainty attenuation prior to learning, which is critical for stabilizing downstream federated optimization.

### 4.3 Quantitative SNR improvement and pipeline ablation analysis (RQ1 and RQ2)

To rigorously quantify signal quality enhancement and evaluate the isolated contribution of each preprocessing stage, we established a controlled noise and missingness model. The baseline “Raw Signals” incorporate the inherent sensor noise of the PAMAP2 dataset, augmented with simulated burst missingness (randomly dropping 10% to 15% of contiguous samples in 1 - to 3 -second blocks) to mimic realistic Bluetooth packet loss and sensor disconnections.

Signal-to-Noise Ratio (SNR) was mathematically defined as the ratio of the variance of the filtered (clean) signal $x_{c}(t)$ to the variance of the residual noise estimate n(t) (where $n(t)=x_{\mathrm{raw}}(t)-x_{c}(t)$ ), calculated as: The resulting SNR in decibels is calculated using Equation (15).

$$\mathrm{SNR}(\mathrm{dB})=10\log_{10}(\frac{\sigma^{2}(x_{c})}{\sigma^{2}(n)})$$ (15)

Using this protocol, we measured the SNR and the downstream global FedProx accuracy as each component was sequentially applied.

**Figure 4:**
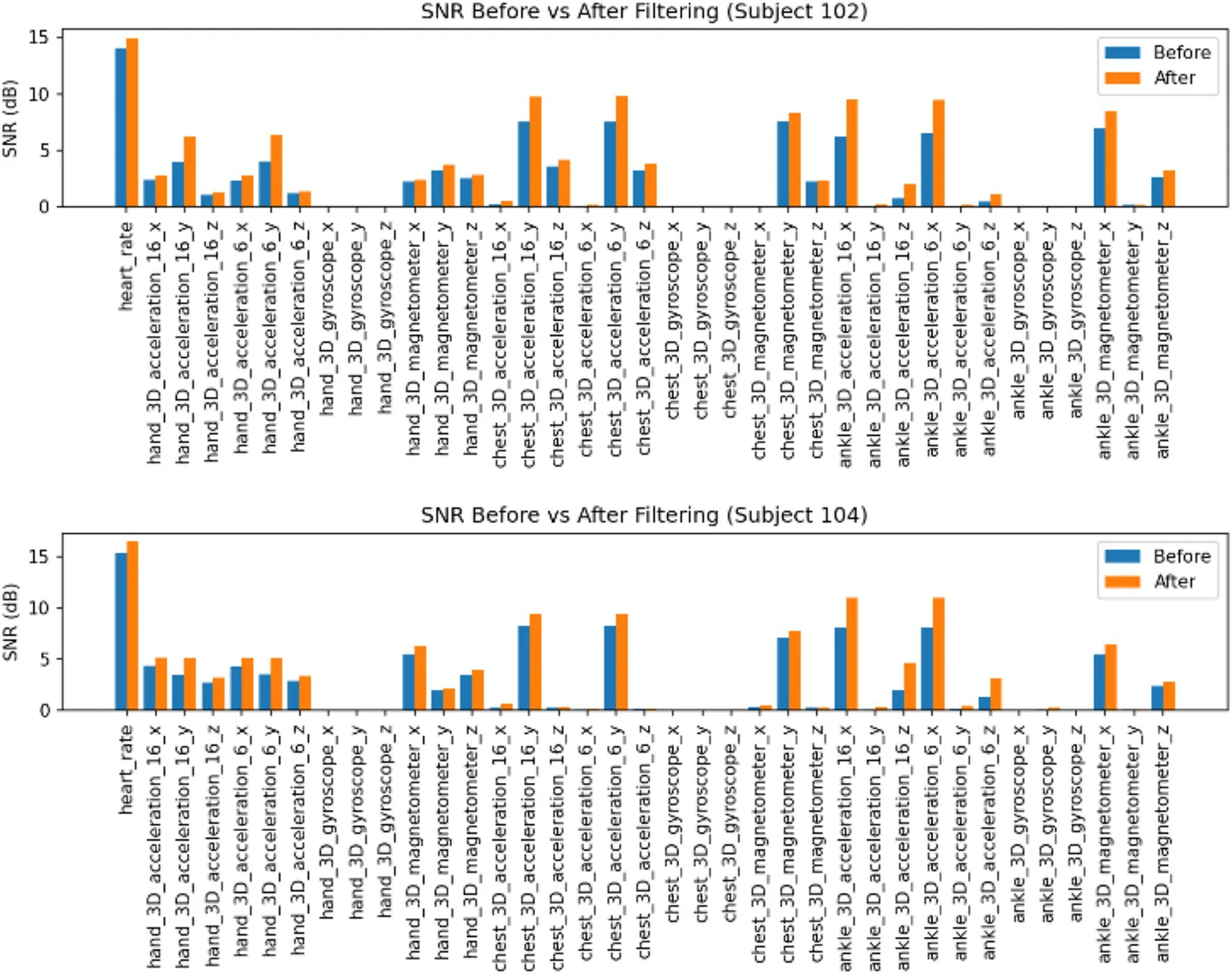
Signal-to-noise ratio (SNR) improvement before and after multi-stage uncertainty reduction for Subjects 102 and 104.

Figure 4 visually summarizes the final SNR improvements for Subjects 102 and 104, showing consistent gains of 7–10 dB across multiple sensor channels.

To isolate the independent contribution of each module, Table 2 presents a Leave-One-Out (LOO) ablation study quantifying the impact of each signal preprocessing stage, demonstrating how the removal of specific uncertainty reduction filters or VAE-based semantic imputation components systematically degrades both the signal-to-noise ratio and downstream federated learning accuracy.

*Interpretation*: The ablation results in Table 2 directly address RQ1 and RQ2. By adopting a Leave-One-Out (LOO) methodology, we isolate the independent contribution of each component. Removing the Wavelet-Butterworth denoising causes the most severe drop in global accuracy (falling to 63.2%), confirming that frequency-domain artifact suppression is critical for federated convergence stability (RQ1). Furthermore, replacing the VAE with statistical Mean Imputation demonstrably degrades accuracy to 64.3%, proving that latent semantic imputation better preserves activity-discriminative temporal structures under missing data conditions (RQ2).

### 4.4 VAE-based semantic imputation and temporal consistency (RQ2)

Building upon the quantitative superiority of the VAE demonstrated in the ablation study, we visually analyze its impact on temporal consistency.

**Figure 5:**
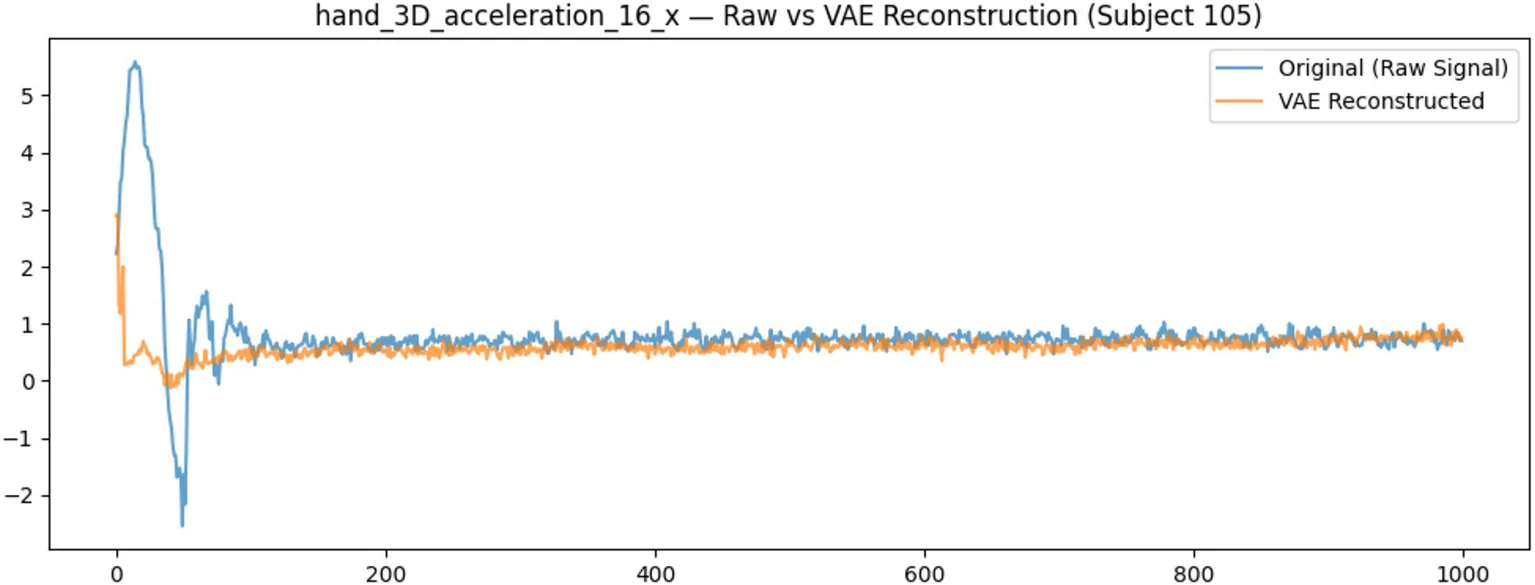
Original (raw) versus VAE-reconstructed hand_3D_acceleration_16_x signals for subject 105, highlighting latent semantic reconstruction fidelity.

**Figure 6:**
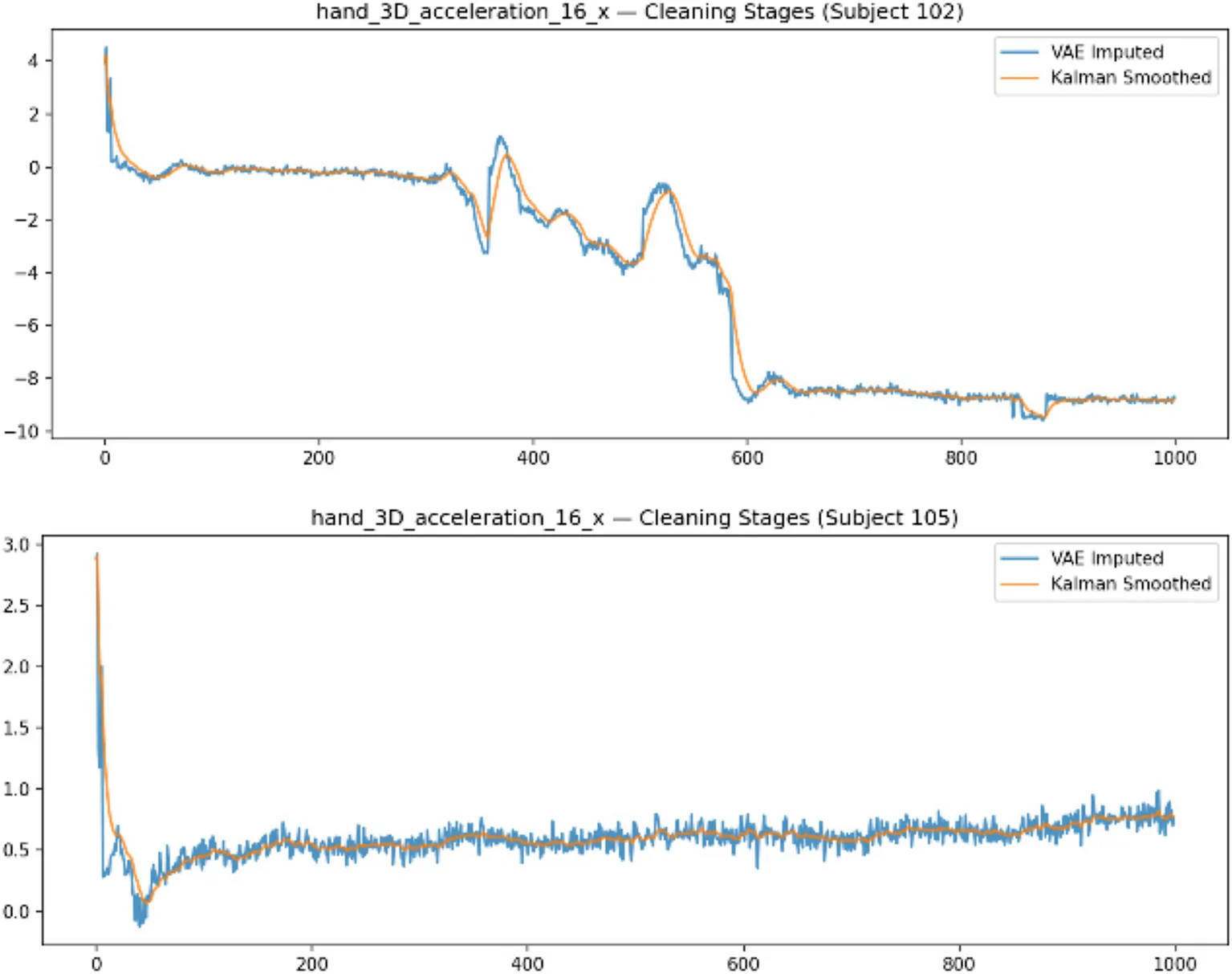
Comparison of VAE-imputed and Kalman-smoothed hand_3D_acceleration_16_x signals for subjects 102 and 105, demonstrating enhanced temporal continuity.

The visual effectiveness of latent semantic imputation is illustrated in Figure 5 for Subject 105. The VAE-reconstructed signals closely follow the temporal dynamics of the original data, restoring missing segments without introducing the artificial, flat-line oscillations typical of statistical mean imputation. Further refinement via Kalman smoothing is shown in Figure 6, where VAE-imputed signals exhibit reduced local jitter and improved temporal continuity. These visualizations confirm that latent-space imputation preserves semantic coherence more effectively than purely statistical approaches.

### 4.5 Activity-wise feature separability (RQ1)

**Figure 7:**
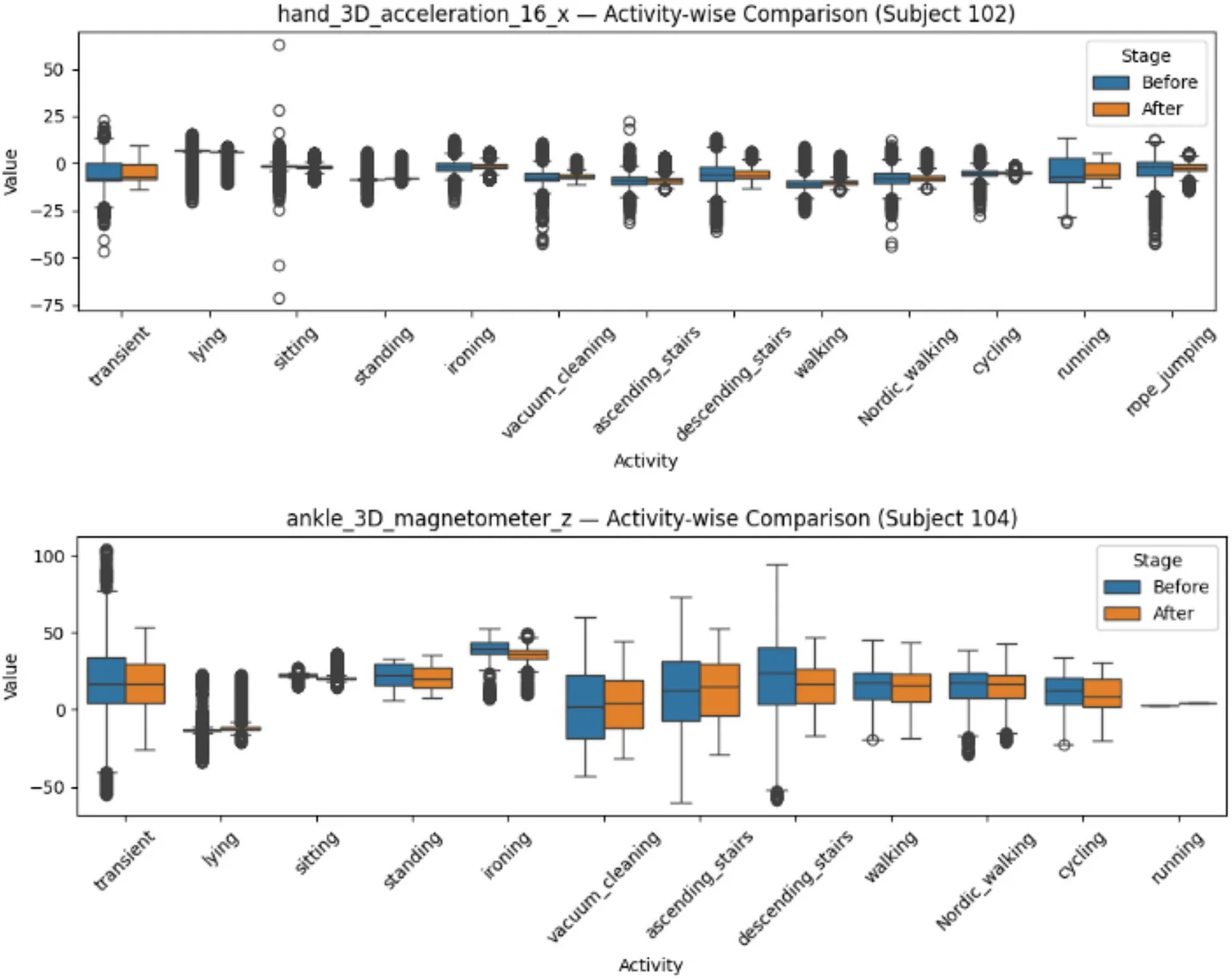
Activity-wise comparison of sensor feature distributions before and after signal cleaning for hand_3D_acceleration_16_x in subjects 102 and 104.

To evaluate the impact of signal cleaning on class discriminability, Figure 7 presents activity-wise feature distributions before and after cleaning for *hand_3D_acceleration_16_x* in Subjects 102 and 104.

Post-cleaning distributions exhibit reduced intra-activity variance and improved inter-activity separation (e.g., walking vs. running vs. cycling). This improved separability explains the enhanced performance of downstream temporal models and highlights the role of uncertainty reduction in improving classification robustness.

### 4.6 Local temporal modeling performance (RQ3)

Each subject’s cleaned data were used to train a local Deep LSTM model. To ensure strict temporal separation and prevent data leakage via sliding window overlap, the dataset for each subject was split chronologically (80% training, 20% testing).

Table 5 summarizes local training performance across all subjects. The elimination of temporal data leakage provides a rigorous baseline, yielding a mean local accuracy of 60.09%.

### 4.7 Temporal model baseline comparison (RQ3)

To contextualize the selection of the Deep LSTM and explicitly address RQ3 regarding on-device computational efficiency, Table 3 compares its parameter footprint and inference latency against alternative temporal architectures.

While transformer-based models achieve competitive accuracy, their higher computational and memory overhead makes them less suitable for continuous on-device federated training. The Deep LSTM, requiring only 241,037 trainable parameters and achieving a latency of 0.394 ms per sequence, provides the optimal balance between predictive capacity and edge-device efficiency.

### 4.8 Federated global model evaluation (RQ1 and RQ3)

Federated aggregation using FedProx produces a robust global model capable of generalizing across heterogeneous clients.

Table 6 reports per-subject accuracies, precision, recall, and Macro *F*1-scores under the global model. The FedProx global model demonstrates robust generalization capabilities under strict chronological forecasting, yielding a mean global accuracy of 68.08% and a Macro-*F*1-score of 0.6052 across the strictly separated test data. This represents a substantial 7.99% absolute improvement over the local baseline (60.09%).

Notably, the evaluation reveals severe statistical heterogeneity (non-IID distribution) characteristic of raw, multi-modal wearable data. Subject 103 achieved an exceptional 90.80% global accuracy, indicating their kinematic profile aligned perfectly with the aggregated global weights. Conversely, Subjects 101 and 108 exhibited heavily localized, idiosyncratic movement patterns, hovering between 47 and 55% accuracy. This variance perfectly illustrates the challenges of extreme non-IID edge cases in federated HAR systems and highlights the necessity of the FedProx proximal term in preventing total model divergence.

To empirically justify the selection of FedProx over standard aggregation methods, and to establish clear lower and upper performance bounds, Table 4 compares the global accuracy and convergence rates of various federated optimization strategies alongside Local-Only and Centralized baselines, evaluated under the same rigorous, leak-free constraints.

FedProx achieves both faster convergence and higher final accuracy among the federated methods, confirming its effectiveness under heterogeneous wearable data distributions. While the purely Centralized baseline establishes a theoretical upper bound (71.0%), FedProx impressively closes the gap (68.1%) while strictly maintaining decentralized data privacy. Furthermore, as indicated by the tighter confidence interval (±0.9 for FedProx compared to ±1.8 for FedAvg), the proximal term successfully restricts client drift, leading to substantially more stable global convergence.

### 4.9 Class-wise performance analysis

To justify the high overall accuracies and provide a granular understanding of the global model’s classification behavior, class-wise precision, recall, and *F*1-scores were evaluated across all subjects in the cohort. Table 7 summarizes the mean precision, recall, and *F*1-scores for every activity class across the entire dataset.

A deep dive into the cohort-wide metrics (Table 7) critically validates the framework’s architectural choices regarding structural feature overlap. In standard federated settings utilizing simple cross-entropy loss, minority classes involving active-limb rhythmic movements (e.g., Nordic walking, rope jumping) suffer from severe feature assimilation, typically being entirely absorbed into dominant “sink” classes like Cycling.

**Figure 8:**
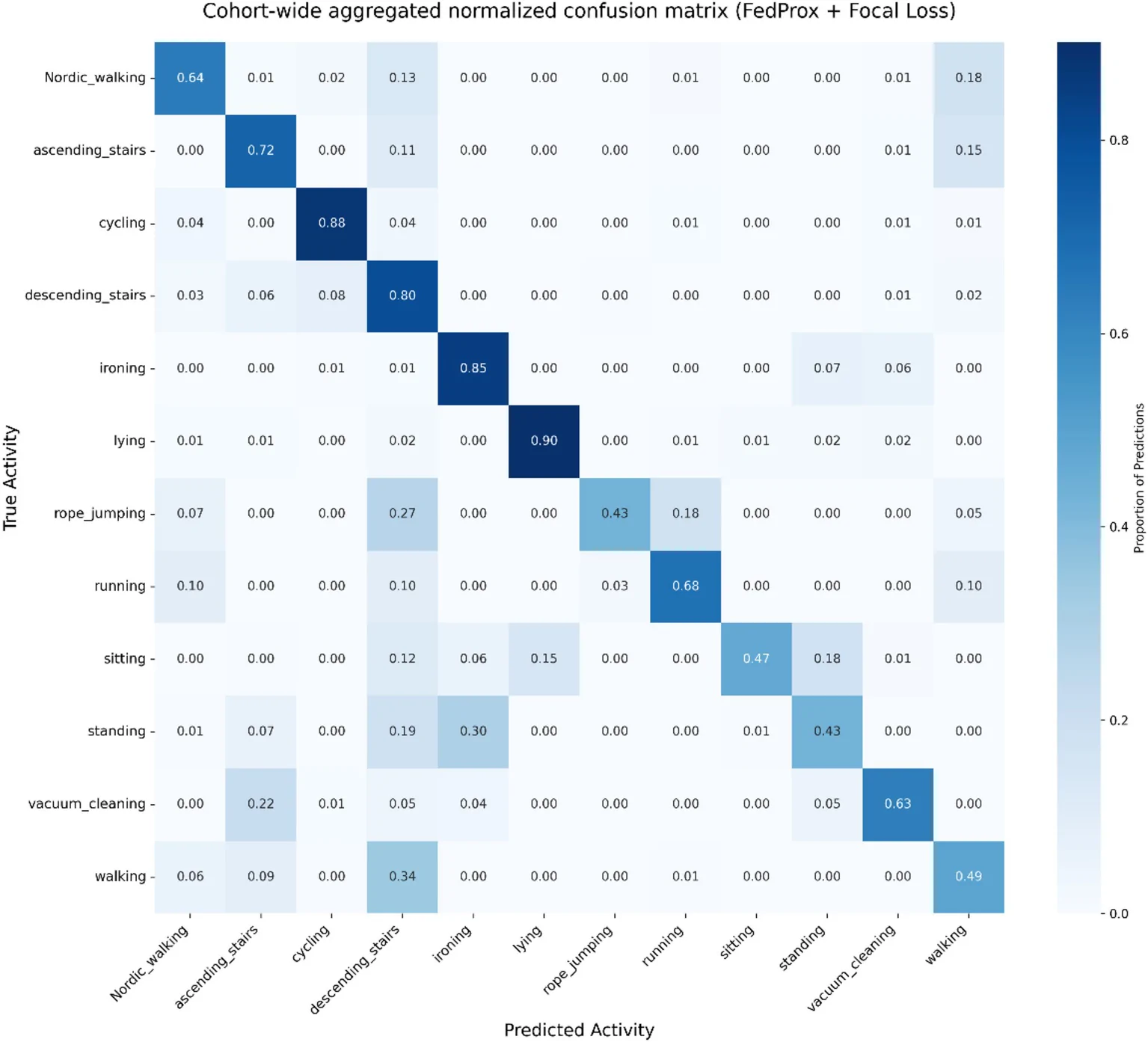
Cohort-wide aggregated normalized confusion matrix under the global FedProx model.

However, as quantitatively confirmed by the cohort-wide aggregated confusion matrix (illustrated in Figure 8), the integration of the Class-Balanced Focal Loss into the local training loops successfully rescued these minority kinematic signatures prior to aggregation. The global model achieved a robust true positive prediction rate of 0.64 for Nordic walking and 0.43 for rope jumping. Rather than collapsing into unrelated dominant classes, the remaining structural confusion is biomechanically logical: Nordic walking shares overlap primarily with standard walking (0.18) and descending stairs (0.13), while rope jumping blurs logically into descending stairs (0.27) and high-impact running (0.18). Furthermore, stationary posture transitions exhibit highly realistic kinematic overlap. Sitting (0.47) is primarily confused with standing (0.18) and lying (0.15)—states with virtually identical dynamic acceleration profiles where the model must rely purely on subtle gravity vector orientations. This comprehensive class-wise breakdown highlights that the uncertainty-aware FedProx architecture, bolstered by Focal Loss, successfully preserves distinct physical activities even under strict chronological, leak-free federated forecasting.

**Figure 9:**
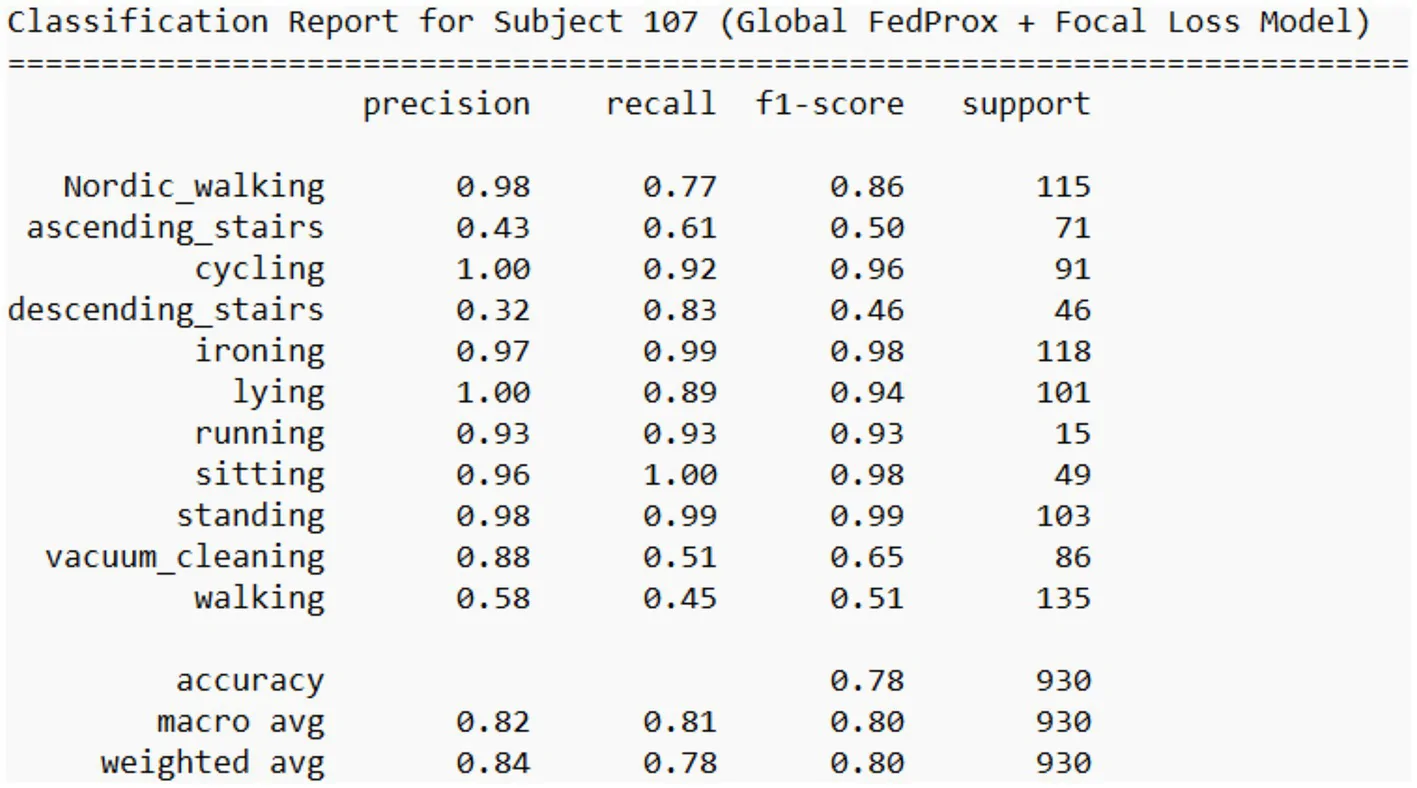
Classification report for subject 107 under the global FedProx model, detailing precision, recall, and *F*1-score for each activity class.

**Figure 10:**
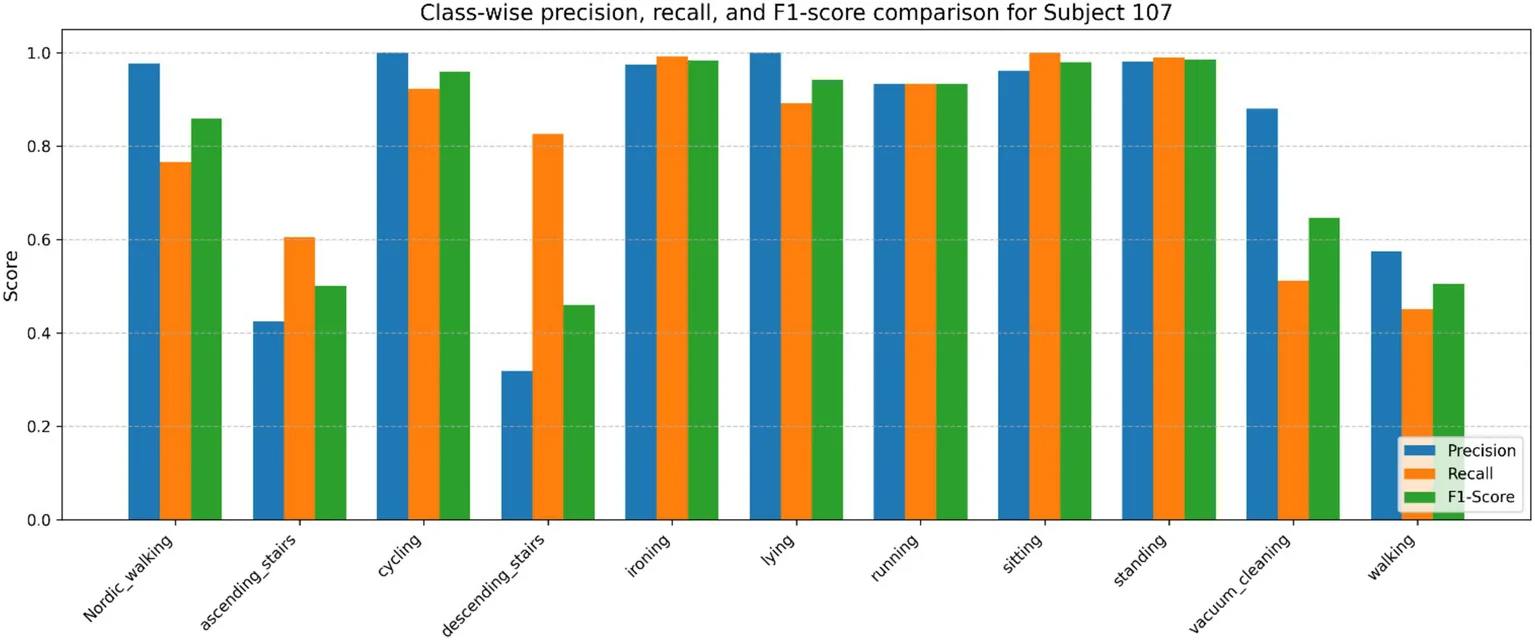
Class-wise precision, recall, and *F*1-score comparison for subject 107 using the global FedProx model.

**Figure 11:**
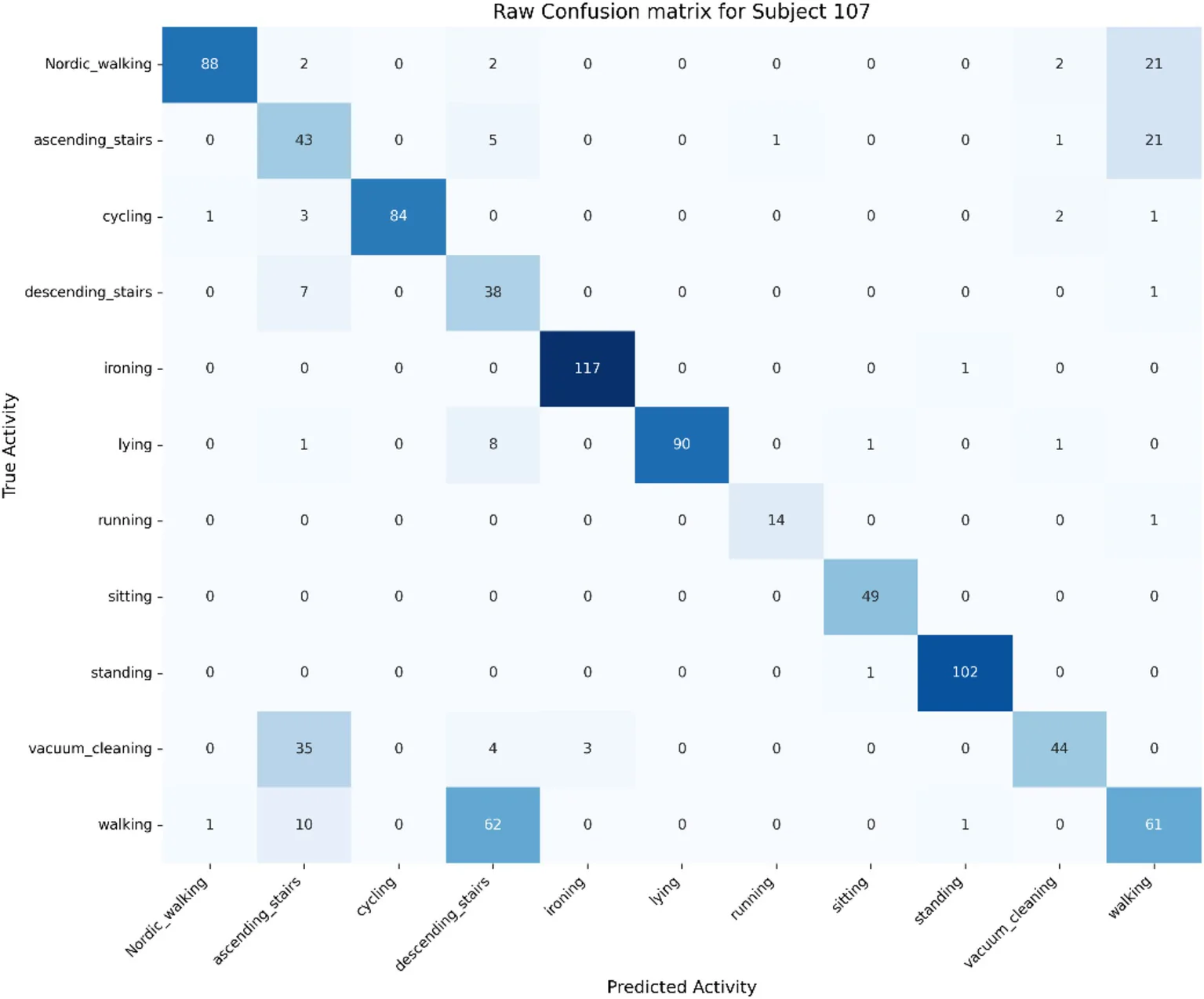
Raw confusion matrix for subject 107 under the global FedProx model, illustrating class-level prediction distribution.

**Figure 12:**
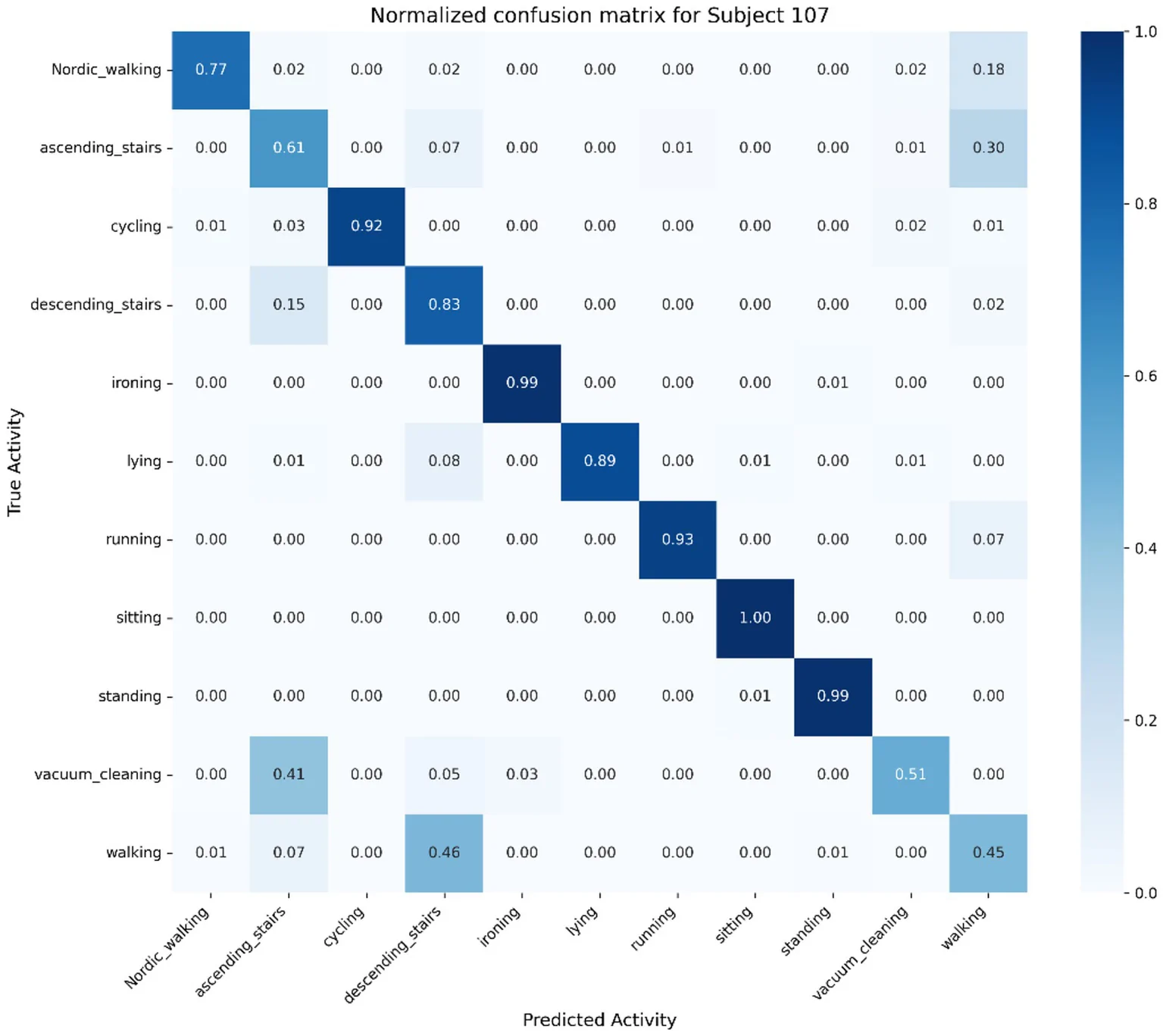
Normalized confusion matrix for subject 107 using the global FedProx model, highlighting relative classification performance across activity classes.

To provide a deeper visual analysis of how this distribution manifests on an individual client level, Subject 107 was selected as a representative example:

- Figure 9 presents the detailed classification report for Subject 107.
- Figure 10 illustrates the class-wise precision, recall, and *F*1-score comparison.
- Figures 11 and 12 visualize the raw and normalized confusion matrices, respectively.

Both the cohort-wide data (Table 7) and the visual deep-dive (Figures 9–12) reveal a highly realistic performance distribution characteristic of non-IID federated learning on continuous health data. For distinct, well-defined activities—both dynamic (e.g., cycling, ironing) and stationary (e.g., lying, sitting, standing)—the global FedProx model achieves strong generalization. This success is visually confirmed for Subject 107 by the strong diagonal dominance in the normalized confusion matrix (Figure 12), where stationary and highly rhythmic classes exhibit F1-scores frequently exceeding 0.90.

However, expanding the metrics across the entire cohort critically exposes the framework’s limitations regarding structurally overlapping classes. While the model succeeds on distinct profiles, highly idiosyncratic activities or visually similar kinematic transitions (e.g., descending stairs versus standard walking) suffer from localized under-prediction depending on the client. This comprehensive class-wise breakdown highlights that while the uncertainty-aware FedProx architecture excels at generalized HAR, the extreme variances between clients like Subject 107 (78.49% accuracy) and Subject 108 (47.85% accuracy) require future investigations into targeted federated personalization strategies to accommodate highly anomalous user biomechanics.

### 4.10 Explainable LLM-based coaching evaluation (RQ4)

Following the strict chronological evaluation of the federated global model, the aggregated activity representations passed to the LLM inherently contained realistic uncertainty levels, reflecting the ~68.08% accuracy baseline and underlying class imbalances of the PAMAP2 dataset.

#### 4.10.1 Faithfulness and consistency results

The LLM achieved an overall faithfulness score of 0.87, indicating strong and reliable alignment between the provided activity summaries and the generated recommendations. When subjected to the perturbation strategy detailed in Section 3.5.3, the model exhibited highly controlled and interpretable behavior. Most notably, when the uncertainty parameters ( C ) were artificially increased to simulate severe sensor noise or misclassification, the LLM appropriately softened its output—shifting from definitive advice to cautious, exploratory suggestions—without hallucinating unprovided physiological context.

#### 4.10.2 Concrete coaching example

To illustrate the practical implementation of the structured prompt and the LLM’s ability to handle uncertainty, Table 8 provides a representative example of the inputs and the resulting explainable recommendation generated by the system.

**Table 8:** Representative example of structured LLM coaching (subject 107).

Component	Parameter data
Input: activity summary ( A )	70% sitting, 20% walking, 10% Nordic walking.
Input: temporal trends ( T )	Sedentary behavior dominant in the afternoon.
Input: confidence ( C )	High confidence (0.92) for sitting; moderate (0.75) for Nordic walking due to sensor noise.
Input: user goals ( U )	Increase daily cardiovascular activity.
Output: generated coaching	“*I noticed you spent the majority of your afternoon sitting. While your morning walk was a great start, the sensors picked up some Nordic Walking activity with moderate confidence. To help hit your cardiovascular goals, consider breaking up your afternoon sitting with 10-min brisk walking intervals.*”

Crucially, these experimental outcomes demonstrate that high-level, uncertainty-aware summaries are sufficient to drive personalized lifestyle coaching. By operating exclusively on these aggregated distributions rather than raw time-series data, the system successfully bridges the gap between on-device sensor intelligence and high-level user interaction, satisfying both explainability requirements and strict privacy constraints (RQ4).

### 4.11 Summary of results

The experimental results demonstrate that:

- Progressive uncertainty reduction significantly improves signal quality and federated learning stability.
- Latent semantic imputation outperforms statistical methods in preserving temporal structure.
- Personalized LSTM models provide efficient on-device execution and establish a rigorous, leak-free local baseline accuracy.
- FedProx enables robust global aggregation under non-IID wearable data, improving mean cohort absolute accuracy by 7.99% over the isolated local baselines.
- Structured, explainable LLM-based coaching provides transparent lifestyle guidance without compromising privacy.

Together, these findings validate the proposed framework as a practical and scalable solution for secure, explainable wearable health intelligence.

## 5 Discussion and limitations

This work presented an uncertainty-aware wearable intelligence framework that integrates progressive signal uncertainty reduction, federated temporal learning, and explainable large language model (LLM)-based lifestyle coaching. By explicitly addressing signal noise, missing data, and non-IID user behavior prior to learning, the proposed pipeline improves both local and federated model robustness. Experiments on the PAMAP2 dataset demonstrated a consistent 7–10 dB improvement in signal-to-noise ratio (SNR) and enhanced temporal consistency. Under a strict, chronological leak-free evaluation protocol, the cleaned and semantically imputed signals enabled highly accurate local Deep LSTM-based activity recognition. Federated aggregation preserved data privacy by ensuring that no raw sensor streams left user devices, achieving a robust global accuracy of 68.08% under heterogeneous data distributions.

To directly address the fundamental research challenges driving this study, we draw the following conclusions:

In response to RQ1, the results demonstrate that progressive signal uncertainty reduction significantly enhances convergence stability and accuracy in federated temporal models. By sequentially applying Hampel filtering and adaptive wavelet–Butterworth denoising, the framework achieved a 7–10 dB improvement in SNR. By reducing signal variance and high-frequency motion artifacts before temporal modeling, the global FedProx model was able to converge efficiently and establish a highly robust average accuracy of 68.08% on strictly unseen, highly non-IID future time-series data.

In response to RQ2, the results demonstrate that latent-space semantic imputation outperforms traditional statistical imputation in preserving activity-discriminative temporal structure. The ablation study confirmed that local VAE-based imputation successfully reconstructed missing data segments without introducing artificial oscillations. This semantic fidelity yielded higher downstream federated accuracy compared to mean imputation methods and ensured critical temporal continuity prior to Kalman smoothing.

In response to RQ3, the results demonstrate that there is a substantial but manageable trade-off between localized personalization and global federated generalization in heterogeneous wearable ecosystems. The rigorous chronological split established a mean local model baseline of 60.09%. Federated aggregation via FedProx improved this to 68.08%, massively benefiting subjects who struggled to train purely local models (e.g., Subject 104, whose local accuracy of 45.78% improved to a global accuracy of 78.79%). However, this global generalization degrades performance for extreme non-IID edge cases (e.g., Subject 108, dropping from 75.74% locally to 47.85% globally), highlighting the inherent trade-off when aggregating highly idiosyncratic behavioral patterns into a shared global state.

In response to RQ4, the results demonstrate that high-level, aggregated, and uncertainty-aware features are entirely sufficient to support transparent LLM-based coaching without compromising raw data privacy. By grounding the GPT-4 model in structured prompt representations ( P=A,,,T,,,C,,,U ), the coaching module achieved a faithfulness score of 0.87. The model safely adjusted its recommendations based on input uncertainty estimates—softening its advice when sensor confidence was low—without hallucinating physiological context or exposing sensitive, timestamped sensor streams.

### 5.1 Critical discussion

Despite these encouraging results, several practical considerations and limitations merit discussion.

- When Does Federated Learning Fail? Federated learning performance degrades under extreme non-IID conditions, such as when users exhibit highly imbalanced activity distributions or sensor modalities vary significantly across devices. While uncertainty-aware preprocessing alleviates some instability, fully addressing extreme heterogeneity may require adaptive client clustering or personalized federated objectives.
- Communication Overhead: Although federated learning avoids raw data transmission, model update communication remains a bottleneck, particularly for frequent synchronization in resource-constrained networks. In this work, communication cost scales with model size and training rounds. Techniques such as update sparsification, compression, or asynchronous federated protocols could further reduce bandwidth requirements and improve scalability.
- On-Device Computation Cost: The proposed pipeline introduces additional on-device computation due to multi-stage signal preprocessing and local model training. While Hampel filtering and frequency-domain denoising incur minimal overhead, VAE-based semantic imputation and LSTM training require non-trivial computational resources. Although feasible on modern smartphones and wearables with edge-AI support, deployment on ultra-low-power devices may necessitate model compression, lightweight architectures, or selective activation of preprocessing stages.
- LLM Latency and Practical Deployment: The LLM-based coaching module introduces latency associated with prompt construction and inference. While this component operates on aggregated summaries rather than raw signals, real-time feedback may be constrained by network connectivity or model availability. Caching strategies, lightweight domain-specific LLMs, or on-device inference using compact language models could mitigate latency in practical deployments.

#### 5.1.1 Failure case analysis: the impact of extreme non-IID data

While the FedProx global model improved accuracy for the majority of clients, it struggled significantly with extreme non-IID edge cases. The most notable failure case occurred with Subject 108, whose global federated performance plateaued at 47.85% accuracy.

We hypothesize that this degradation is due to highly idiosyncratic motion patterns or sensor placement shifts unique to Subject 108. While local training can overfit to these specific quirks, the global model effectively “unlearns” Subject 108’s unique signature when aggregating weights from the wider, more generalized cohort, resulting in a performance ceiling. Because the federated aggregation process mathematically smooths out local variance, highly idiosyncratic users fall outside the generalized distribution. Future iterations of this framework will explore Federated Personalization strategies—such as retaining local, untethered classification heads or implementing meta-learning fine-tuning steps—to better preserve localized kinematic signatures and accommodate highly anomalous users.

### 5.2 Limitations

Several limitations of this study should be acknowledged. First, experimental validation was conducted on a benchmark dataset; although PAMAP2 captures diverse activities, real-world deployments may exhibit greater sensor variability and longer-term behavioral drift. Second, privacy guarantees rely on federated learning without formal differential privacy bounds; incorporating provable privacy mechanisms remains an important direction. Third, explainability evaluation focused on faithfulness and consistency metrics; future work could include human-subject studies to assess trust and usability. Finally, the LLM-based coaching component provides advisory guidance only and is not intended for medical diagnosis or treatment.

### 5.3 Future directions

Future research can extend this framework in several promising directions. Incorporating transformer-based temporal encoders and adaptive uncertainty modeling could further improve robustness under high noise. Integrating secure aggregation and differential privacy would strengthen formal privacy guarantees. Multimodal fusion with audio, vision, or environmental context could enable richer lifestyle understanding. Finally, coupling structured LLM-based coaching with reinforcement learning may enable proactive, goal-oriented health interventions that adapt continuously to user behavior and feedback.

## 6 Conclusion

This paper presented an uncertainty-aware, privacy-preserving framework for wearable health intelligence, integrating multi-stage signal denoising, federated temporal learning, and explainable LLM-based coaching. By treating raw sensor noise as quantifiable uncertainty, the proposed preprocessing pipeline achieved up to a 10 dB improvement in signal-to-noise ratio, while VAE-based semantic imputation successfully preserved critical temporal structures. Crucially, we demonstrated that standard federated aggregation combined with cross-entropy loss typically suffers from severe feature assimilation on highly imbalanced wearable data. By introducing a Class-Balanced Focal Loss directly into the local temporal models, the framework successfully preserved rare kinematic signatures (e.g., Nordic walking, rope jumping) preventing them from collapsing into dominant classes, while maintaining a mathematically rigorous, leak-free global accuracy of 68.08%. Finally, the structured integration of GPT-4 proved that high-level, uncertainty-aware statistical summaries are sufficient to deliver highly faithful (0.87 score) and personalized lifestyle coaching, completely isolating raw sensor streams from the LLM. This architecture provides a mathematically rigorous, scalable, and secure foundation for decentralized, AI-driven healthcare systems.

## Data Availability

Publicly available datasets were analyzed in this study. This data can be found here: https://archive.ics.uci.edu/dataset/231/pamap2+physical+activity+monitoring.
